# Age dependency of trauma-induced neocortical epileptogenesis

**DOI:** 10.3389/fncel.2013.00154

**Published:** 2013-09-18

**Authors:** Igor Timofeev, Terrence J. Sejnowski, Maxim Bazhenov, Sylvain Chauvette, Laszlo B. Grand

**Affiliations:** ^1^Department of Psychiatry and Neuroscience, Université LavalQuébec, QC, Canada; ^2^Le Centre de Recherche de l’Institut Universitaire en santé Mentale de QuébecQuébec, QC, Canada; ^3^Computational Neurobiology Laboratory, Howard Hughes Medical Institute, The Salk Institute for Biological StudiesLa Jolla, CA, USA; ^4^Division of Biological Sciences, University of California at San DiegoLa Jolla, CA, USA; ^5^Department of Cell Biology and Neuroscience, University of California at RiversideRiverside, CA, USA

**Keywords:** sleep, wake, trauma, excitability, epileptogenesis, seizure, epilepsy

## Abstract

Trauma and brain infection are the primary sources of acquired epilepsy, which can occur at any age and may account for a high incidence of epilepsy in developing countries. We have explored the hypothesis that penetrating cortical wounds cause deafferentation of the neocortex, which triggers homeostatic plasticity and lead to epileptogenesis (Houweling etal., 2005). In partial deafferentation experiments of adult cats, acute seizures occurred in most preparations and chronic seizures occurred weeks to months after the operation in 65% of the animals (Nita etal., 2006,^2007^; Nita and Timofeev, 2007). Similar deafferentation of young cats (age 8–12 months) led to some acute seizures, but we never observed chronic seizure activity even though there was enhanced slow-wave activity in the partially deafferented hemisphere during quiet wakefulness. This suggests that despite a major trauma, the homeostatic plasticity in young animals was able to restore normal levels of cortical excitability, but in fully adult cats the mechanisms underlying homeostatic plasticity may lead to an unstable cortical state. To test this hypothesis we made an undercut in the cortex of an elderly cat. After several weeks this animal developed seizure activity. These observations may lead to an intervention after brain trauma that prevents epileptogenesis from occurring in adults.

## INTRODUCTION

Epilepsy is used to describe over 40 different types of neurological pathologies resulting from different etiologies. The main common features of epilepsy are the presence of unprovoked seizures and the abnormal local neuronal synchronization (Timofeev et al., 2012). Traumatic brain injury in particular is a major risk factor for epileptogenesis (Feeney and Walker, 1979; Temkin et al., 1995; Annegers et al., 1998). Cortical trauma leads to paroxysmal activity within 24 h in up to 80% of patients with penetrating wounds and stops within a 48 h period (Kollevold, 1976; Dinner, 1993). In Vietnam and Croatia, post-war epidemiological studies reported that about 50% of patients with penetrating cranial wounds develop recurring seizures 10–15 years after the trauma (Salazar et al., 1985; Marcikic et al., 1998).

**FIGURE 1:**
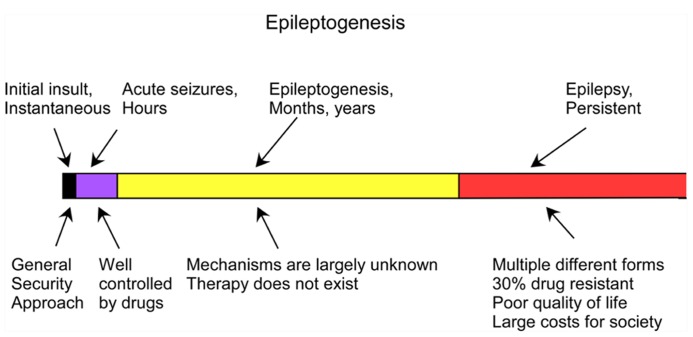
**Time course of the development of epilepsy from brain trauma (adapted from Timofeev, 2011)**.

Trauma-induced epilepsy is poorly controlled by the currently available medication. Early administration of anticonvulsant medication decreases the percentage of early posttraumatic seizures but does not prevent chronic epilepsy (Temkin et al., 1990, 1999; Chang and Lowenstein, 2003). Thus, understanding the mechanisms of trauma-induced epileptogenesis (TIE) – the set of latent processes caused by the initial insult that lead to the development of epilepsy – may lead to the development of new preventive approaches (**Figure 1**).

Both the cortex and the underlying white matter are damaged in the vast majority of brain-penetrating wounds. We review what is known about the changes that occur in the cortex following brain trauma. Experiments with direct damage of the cortex and the underlying white matter in young and adult cats will be described in detail below. The evidence points toward homeostatic mechanisms that may account for the differences between the effects of brain trauma in young and adult cats.

## CORTICAL ACTIVITY DURING STATES OF VIGILANCE

There are three major states of vigilance: waking (W), slow-wave sleep (SWS), and rapid eye movement (REM) sleep. During normal levels of cortical activity, excitation and inhibition are balanced. At the level of neocortex, persistent synaptic activity and neuronal firing characterize both waking state and REM sleep, but in contrast, during SWS the membrane potential oscillates between depolarized and hyperpolarized states. Changes in the state of vigilance are controlled by shifts in the levels of neuromodulation.

During waking state, electroencephalography (EEG) activities are characterized by low-amplitude, high-frequency oscillations in the beta and gamma frequency ranges in a majority of cortical regions (Niedermeyer, 2005). In this state, the membrane potential of cortical neurons is relatively depolarized (around -62 mV), display continuous excitatory and inhibitory synaptic activity, and spontaneously fire action potentials (Timofeev et al., 2000, 2001; Steriade et al., 2001; Mukovski et al., 2007; Rudolph et al., 2007). During quiet wakefulness, an 8–12 Hz alpha rhythm is generated over the visual cortex in awake human subjects with closed eyes (Compston, 2010) and an 8–12 Hz mu rhythm is recorded over the somatosensory cortex during immobility (Rougeul et al., 1972; Rougeul-Buser et al., 1975; Bouyer et al., 1983). Because of their relatively high amplitude, neurons contributing to these rhythms are likely to have synchronous membrane potential fluctuations in the same frequency bands. Indeed, whole-cell patch recordings from neurons in the barrel cortex of mice have large, coordinated amplitude fluctuations of the membrane potential prior to and following whisking, but these fluctuations were dramatically reduced during whisking (Crochet and Petersen, 2006; Poulet and Petersen, 2008; Gentet et al., 2010). These fluctuations appear to be slower than the mu rhythm recorded in humans and cats, but faster than sleep slow oscillation recorded in mice (Fellin et al., 2009). Recordings from cell bodies demonstrated that coordinated inhibitory activity dominated during quiet wakefulness, in responses to visual stimuli, as well as in other states of vigilance (Rudolph et al., 2007; Haider et al., 2013).

The main electrographic characteristic of SWS is the presence of slow rhythmic activity (Blake and Gerard, 1937), which includes not only slow waves composing the core of the slow oscillation (Steriade et al., 1993), but also spindles and faster activity including beta oscillations, gamma oscillations, and ripples grouped by the slow oscillation (Steriade, 2006). Intracellular recordings during SWS demonstrate that depth-positive (surface-negative) waves of local field potential (LFP) are accompanied with periods of disfacilitation, which results in significant hyperpolarization and silence of cortical neurons with dramatically reduced or abolished synaptic activity. During depth-negative (surface-positive) waves, cortical neurons display intense synaptic activity, neuronal depolarization, and mean membrane potentials similar to those recorded in quiet wakefulness states (Timofeev et al., 2000, 2001; Steriade et al., 2001). In intracellular recordings from non-anesthetized rats, when slow/delta frequencies ranges were present in the LFP, nearby neurons also displayed synchronous alternation of de- and hyperpolarizing states. When the LFP was more activated, neurons displayed relatively stable values of membrane potential and the LFP–intracellular synchrony was decreased (Okun et al., 2010). The amplitude of slow waves recorded intracellularly is quite large (around 10 mV) and similar in different cortical regions in anesthetized animals. During sleep there are area-dependent differences between cortical areas and the intracellular slow-wave amplitude in motor and somatosensory areas is lower but higher in visual and associative areas (Chauvette et al., 2011). Interestingly, in sleep-deprived rats isolated slow waves accompanied with silencing of neuronal firing can be found in otherwise awake behaving animals (Vyazovskiy et al., 2011). In adult human brain, slow-wave activity originate in frontal (Massimini et al., 2004) or medial prefrontal cortex (Nir et al., 2011). However, in young children the slow-wave activity is more intense over occipital cortex, shifting to parietal areas in adolescents and becoming stronger in frontal cortex in adults (Kurth et al., 2010). During aging, slow-wave activity decreases in all cortical regions and this decrease is more pronounced in males than in females (Carrier et al., 2011). Neuronal recordings from cortical slices maintained *in vitro* (Sanchez-Vives and McCormick, 2000), as well as from multisite intracellular recordings *in vivo* (Chauvette et al., 2010) in response to optogenetic stimulation (Beltramo et al., 2013; Stroh et al., 2013) demonstrated that the activity starts preferentially in cortical layer V. However, experiments on epileptic patients suggest that superficial cortical layers play a leading role in the generation of spontaneous cortical active states (Cash et al., 2009; Csercsa et al., 2010).

Rapid eye movement sleep is characterized by activated EEG, complete disappearance of muscle tone, and rapid ocular movements (Luppi et al., 2013). Similar to waking state the membrane potential of cortical neurons is relatively stable, depolarized, and neurons fire spontaneous action potentials (Steriade et al., 2001; Timofeev et al., 2001).

## CORTICAL ACTIVITY DURING STATES OF EPILEPSY

Multiple brain structures are involved in seizure generation. In neocortical epilepsy the neocortex is a primary source of epileptic activity (Timofeev, 2010). Neocortical seizures that are primarily focal often become secondarily generalized tonic–clonic seizures (Crunelli and Leresche, 2002). Electrographically, these seizures are commonly composed of spike-wave/polyspike-wave EEG discharges at 1.0–2.5 Hz and runs of fast spikes at 7–16 Hz (Timofeev and Steriade, 2004). LFP recordings revealed the presence of ripple activity during spike components of spike-wave complexes, in particular at the onset of electrographic seizures (Grenier et al., 2003). During the spike component of spike-wave complexes, both excitatory and inhibitory cortical neurons are implicated in the generation of paroxysmal depolarizing shift (PDS). Within PDS, a majority of regular-spiking (mainly pyramidal) neurons generates only one or a few spikes, while fast-spiking (mainly inhibitory) interneurons fire throughout PDS with very high frequencies (Timofeev et al., 2002). Therefore, inhibitory activity dominates synaptic components of spikes of spike-wave complexes. Given that during seizure activity the reversal potential for GABA_A_ inhibitory postsynaptic potential (IPSP) is shifted toward more depolarized values (Cohen et al., 2002; Timofeev et al., 2002), the reversed IPSPs contribute to the generation of PDS.

Chemical synaptic interactions might not be the most important mechanism that generates a PDS. During seizures the extracellular concentrations of Ca^2^^+^ decreases and K^+^ increases (Heinemann et al., 1977; Pumain et al., 1983; Somjen, 2002). A reduction in the extracellular Ca^2+^ by itself impairs the presynaptic release of neurotransmitter (Katz, 1969). However, simultaneous reduction in Ca^2+^ and increase in K^+^ in the extracellular milieu to the levels attained during seizure activity also prevents action potential propagation dramatically impairing chemical synaptic interactions (Seigneur and Timofeev, 2010). Therefore mechanisms other than chemical synaptic interactions may be responsible for short-range synchronization, such as electrical coupling via gap junctions, ephaptic interactions, and extracellular communication via activity-dependent ionic changes (Jefferys, 1995; Timofeev et al., 2012). Several research groups have demonstrated on several models of neocortical epilepsy that thalamocortical neurons are not a major contributor to the generation of these cortical seizures (Steriade and Contreras, 1995; Pinault et al., 1998; Timofeev et al., 1998; Steriade and Timofeev, 2001; Meeren et al., 2002; Polack and Charpier, 2006; Polack et al., 2007; Nita et al., 2008b). The runs of paroxysmal fast EEG spikes also have a purely cortical origin since thalamocortical neurons do not show significant oscillatory activity when fast runs occur (Timofeev et al., 1998). At the level of neocortex, fast runs start and terminate almost simultaneously over large distances, suggesting the presence of a common input responsible for turning on and off these fast runs. However, within fast runs, the synchrony is loose; neighboring sites of neocortex (<1 mm inter-electrode distance) can oscillate with different frequencies (Boucetta et al., 2008). The fast-spiking neurons usually oscillate at double the frequency of nearby recorded LFP (Timofeev et al., 1998). These observations suggest that during seizure, long-range (mainly chemical) synaptic interactions do not have a leading role in the synchronization of neuronal activity (Timofeev et al., 2012).

Neocortical seizures are nocturnal, occurring more often during SWS (Timofeev, 2011), and when they occur during SWS, the secondary generalized seizures last much longer than during wake (Bazil and Walczak, 1997). Why should SWS be a factor in the onset of cortical seizures? One of the major differences between SWS and other states of vigilance is the low activity of neuromodulatory systems and, as a result, the network cannot maintain permanent active states. Therefore, the main difference between SWS and other states of vigilance in the cortex is the presence of hyperpolarized silent states.

Several types of anesthesia also create alternating silent and active states. If the anesthetic used does not increase GABAergic processes and does not decrease gap-junction communications, it is often a seizure-triggering factor. In particular, ketamine–xylazine anesthesia in cats induces slow oscillation (alternation of active and silent states). The duration of silent states in anesthetized animals was 150–200% longer than during sleep, depending on the cortical area (Chauvette et al., 2011). As a result, 75% of cats maintained under ketamine–xylazine anesthesia for several hours exhibited electrographic seizures (Boucetta et al., 2008). An increase in network silence may be a factor contributing to seizure onsets because prolonged network silence increases neuronal excitability (reviewed in Timofeev, 2011).

## PARTIAL CORTICAL DEAFFERENTATION IS A MODEL FOR TRAUMA-INDUCED EPILEPTOGENESIS

**FIGURE 2:**
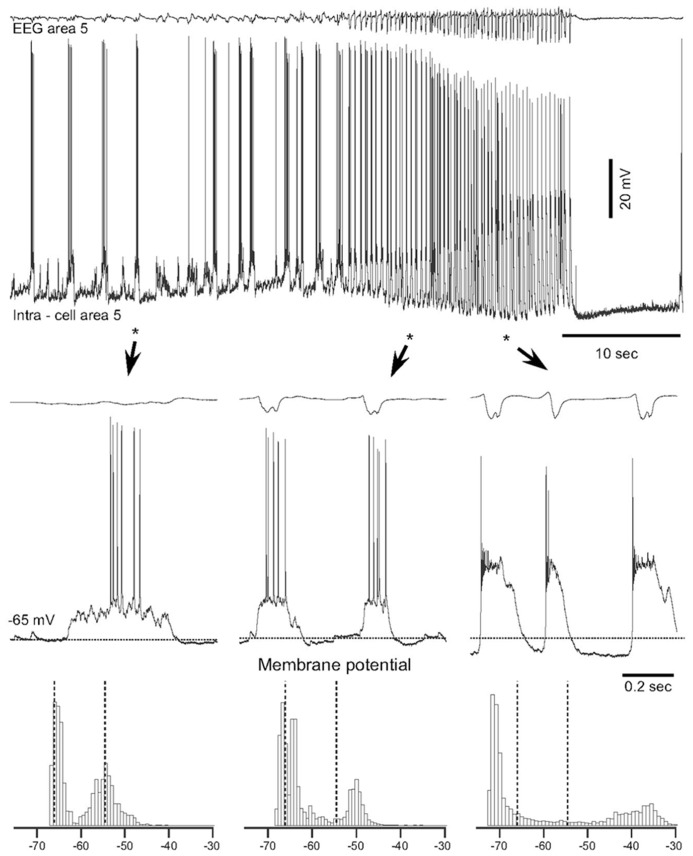
**Spontaneous electrographic seizures in partially deafferented cortex associated with increases in the maximal neural depolarization and hyperpolarization.** Upper panel shows EEG and intracellular recordings from a neuron in area 5, 3 h after the undercut. Three fragments depicted by stars and arrowheads are expanded below. Horizontal dotted lines in middle panels indicate the level of membrane potential (-65 mV). Bottom panels show histograms of the membrane potential (*V*_m_) distribution during corresponding periods of seizure. Dotted lines indicate the initial level of *V*_m_ during the slow oscillation. Note a shift of *V*_m_ during seizure in both depolarizing and hyperpolarizing directions (Topolnik et al., 2003b).

Epilepsy induced by a penetrating wound progresses through the same stages of epileptogenesis as other forms of acquired epilepsy. Multiple forms of TIE has been described, but much less attention was paid to epileptogenesis triggered by penetrating wounds (Hunt et al., 2013). In this experimental model a large part of axons connecting a given cortical area with other brain regions is severed (as example see **Figure 2** in Timofeev et al., 2010). Our previous experiments on cats demonstrated that immediate reaction to brain penetration, in which only slight cortical but large white matter damage was produced, resulted in a dramatic reduction of LFP amplitudes in areas above the damaged white matter. About 3–4 h after the cortical undercut was produced, there were two major outcomes. In 30% of anesthetized cats slow-wave activity was fully or partially recovered. However, in the remaining 70% of animals, slow oscillatory activity was periodically transformed into paroxysmal discharges (Topolnik et al., 2003a, b). An example of electrographic seizure in ketamine–xylazine-anesthetized cat with an undercut cortex is shown in **Figure 2**. The slow oscillation in the undercut cortex is different from the normal slow oscillation: in acute conditions, above the undercut area, silent states last longer than usual (**Figure 2**, left, see also Figure 4 in Topolnik et al., 2003b). The electrographic seizure evolves continuously from the slow oscillation. Seizure onset is characterized by a shortening of both active and silence states and a slight increase in the amplitude of depolarization during active states (**Figure 2**, middle). The body of seizure was associated with a slight, steady hyperpolarization and a dramatic increase in the amplitude during PDS (**Figure 2**, right). Under anesthesia, the seizures usually terminated with postictal depression characterized by EEG flattening and neuronal hyperpolarization (**Figure 2**). This paroxysmal activity usually lasted for 8–10 h and then spontaneously stopped.

**FIGURE 3:**
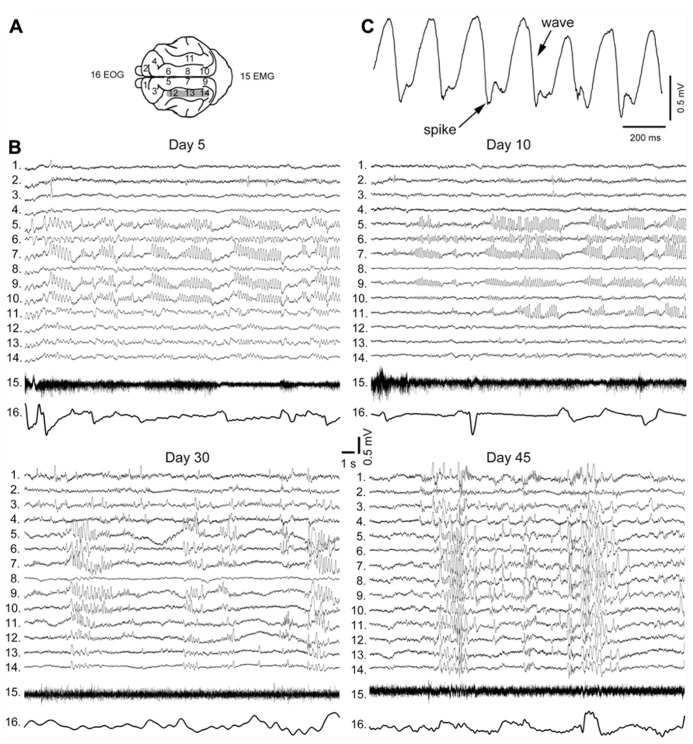
**Electrographic signatures of partial deafferentation-induced epileptogenesis in a cat. (A)** Location of recording electrodes in cats brain. **(B)** Paroxysmal activities recorded at 5, 10, 30, and 45 days post-surgery. Note an increasing with time number of electrodes revealing paroxysmal discharges. **(C)** An expanded segment of paroxysmal discharge showing spike and wave pattern (modified from Nita et al., 2007).

The early stages of seizure activity (0–4 days after undercut) without anesthesia were not investigated previously in the undercut model of epileptogenesis in cats (but see below). At 4–5 days following partial cortical deafferentation, *paroxysmal oscillatory activity was observed in a subset of electrodes surrounding the traumatized area*, but not within undercut (see **Figure 3A** for electrode location) (Nita et al., 2006, 2007). With time, electrographic paroxysmal activity spreads to other cortical regions. In experiment shown in **Figure 3B** during early phases of epileptogenesis (5 days after undercut) the paroxysmal activities occurred in marginal gyrus (electrodes 5, 7, and 9), but not in suprasylvian gyrus (electrodes 12–14) in which the undercut was made. Often paroxysmal discharges occurred in contralateral foci (electrodes 9 and 10), likely due to callosal transmission of synchronous neuronal firing from paroxysmal focus. These paroxysmal discharges were primarily composed of spike-and-wave complexes (**Figure 3C**). Similar pattern was seen in 10 days from the undercut. However, in 30 days from partial deafferentation of suprasylvian gyrus the paroxysmal activities could be detected on multiple electrodes including the undercut cortex. In 1.5–4 months from the deafferentation, when most of the investigated areas revealed periodic paroxysmal discharges (**Figure 3B**), behavioral seizures began in 65% of cats (Nita et al., 2007). Our data indicate that seizures usually invaded the undercut cortex within a month or more, suggesting that axonal sprouting between intact and undercut cortex might play a role in the propagation of seizures. Electrographic seizures were present during waking state, were dramatically enhanced during SWS, but were absent during REM sleep. Neuronal activity during brain activated states in partially deafferented cortex, even outside seizures was different than activity in intact cortex. Similar to intact cortex, during SWS, neurons oscillated between depolarizing and hyperpolarizing states (see above). However, silent (hyperpolarizing) states in partially deafferented cortex were also found during both quiet wakefulness and even REM sleep (Timofeev et al., 2010). These and related data suggest that *prolonged network silence during seizure-free periods is a major contributing factor in TIE* (Timofeev, 2011).

### OTHER MODELS OF EPILEPTOGENESIS

Repetitive brain stimulation induces kindling that eventually leads to the development of spontaneous seizure activity (Goddard, 1967). We recently investigated electrographic activity in cats undergoing neocortical kindling. Repetitive neocortical stimulation was not efficient in generating kindling or seizures if applied during stable states of vigilance. However, when the kindling stimulation was delivered in the first minute of a transition from SWS to wake, it was highly efficient in triggering seizure activity (Nita et al., 2008a, b). After the end of these seizures, cats displayed continuing oscillatory activity with a frequency around 1.5 Hz lasting from tens of minutes and progressively increasing to hours (Nita et al., 2008a). During the continuing seizure activity intracellular recordings from cortical neurons revealed the alternation of active and silent states, although silent states were not accompanied by large amplitude hyperpolarization. Therefore, the overall presence of silence in this cortical network was increased.

Another model for epileptogenesis is chronic GABA infusion, which suppresses activity in the region of infusion. Paroxysmal activity emerges upon withdrawal from chronic GABA infusion (Brailowsky et al., 1988, 1989; Silva-Barrat et al., 1992). The hyperpolarization induced by GABA infusion is consistent with the hypothesis that reduced activity or periodic silence in some cortical territories for days, weeks, or months favor the occurrence of seizures.

### STRUCTURAL CHANGES ACCOMPANYING PARTIAL CORTICAL DEAFFERENTATION

Cutting the majority of fibers arriving to a given region triggers a set of morphological and functional changes. Axonal transection causes some neurons to degenerate and others that survive may exhibit axonal sprouting.

#### Neuronal degeneration

Partial cortical deafferentation produced a dramatic reduction (≈25%) in cortical thickness (Avramescu et al., 2009) suggesting a reduction in the number of cortical cells in the affected region. Detailed examination of the cortex above the white matter transection has demonstrated the presence of delamination, reduction in the number of neurons, shrinkage as well as change in neuronal orientation in cortical depth (Avramescu et al., 2009). These features are similar to those described in the developmental condition called shaken infant syndrome (Marin-Padilla et al., 2002) although mechanisms leading to these changes might be different. The neuronal loss was not equally distributed over the cortical depth profile. There was a major loss of excitatory neurons in deep cortical layers (below 1.5 mm) with a moderate loss in superficial layers. By contrast, there was a highly significant loss of GABAergic neurons in both superficial and deep, but not in middle cortical layers. Why GABAergic neurons were reduced in number is not clear. These cells in the neocortex possess short axons that normally do not reach white matter. It is generally agreed that recurrent seizures may induce neuronal loss in affected brain regions (Cendes, 2005). However, in the cortical undercut model, seizures always started and generally dominated in areas surrounding the undercut cortex (Topolnik et al., 2003b; Nita et al., 2006, 2007) but not within the undercut cortex. In the region surrounding the undercut cortex, there was no significant alteration in the distribution of neuron types as observed within the undercut region (Avramescu et al., 2009). The cause of interneuronal loss remains to be investigated. One of the causes might be a higher dependency of interneurons upon aerobic metabolism than other types of cortical neurons (Sloper et al., 1980; Ribak et al., 1982). Another cause would be a high kainate neurotoxicity of interneurons due to the presence of AMPA/kainate receptors with high calcium permeability (Iino et al., 1990; Weiss et al., 1990). These are possibly the main two factors responsible for preferential loss of GABAergic cells in the undercut model. In layer V pyramidal neurons from the undercut cortex of rats, there was a tendency toward reduction of basal and apical dendritic branches, but these differences did not reach the level of significance (Salin et al., 1995; Avramescu et al., 2009). The preservation of layer V neurons in the undercut model might look surprising, because the axons of these neurons were severed. However, layer V neurons possess very extensive local intracortical connectivity (Markram et al., 1997), which helped them to survive cutting of extracortically going axon. In corticospinal neurons axotomy induced a reduction in the size of cell bodies (Tseng and Prince, 1996).

#### Axonal sprouting

Sometimes severed axons grow to re-innervate the targeted tissue. Axonal damage of corticospinal neurons at the level of the cervical spinal cord, however, did not induce noticeable sprouting within neocortex (Tseng and Prince, 1996). In contrast, a cortical undercut produced local sprouting characterized by increased total axonal length, increase in the number of axonal collaterals and number of axonal swellings (Salin et al., 1995) suggesting that local factors and not the damage of axon per se may trigger the axonal sprouting process. Glutamate uncaging experiments revealed that cortical undercut increased the number of “hot spots” on layer V pyramidal neurons accompanied with a decrease in the amplitude of individual excitatory postsynaptic current (EPSCs; Jin et al., 2006); the increased number of “hot spots” was also found in fast-spiking interneurons (Jin et al., 2011). The level of “inhibitory hot spots” was decreased for both pyramidal cells and interneurons (Jin et al., 2011). Direct investigation of connectivity patterns performed *in vivo* revealed that in partially deafferented cortex the connection probability between neurons was increased starting at 2 weeks after the undercut (Avramescu and Timofeev, 2008). The amplitude of EPSPs in chronic stages was significantly increased compared to controls at 2 and 6 weeks from the undercut, but it was significantly lower at 4 weeks. The coefficient of variation of responses was decreased with time suggesting a more reliable functioning of implicated synapses. It should be noted that synchronous network activity controls axonal sprouting after cortical trauma (Carmichael and Chesselet, 2002). Therefore, cortical trauma induces a pathological loop: axonal sprouting contributes to increased network synchronization leading to seizure and synchronous cortical paroxysmal activity in the partially deafferented cortex directly contributes to the reinforcement of sprouting. Altogether, a partial cortical isolation increases the number and the duration of silent states in the cortical network, which boosts neuronal connectivity and synaptic (network) excitability.

### CHANGES IN THE INTRINSIC EXCITABILITY IN THE PARTIAL CORTICAL DEAFFERENTATION MODEL

Following cortical trauma, intrinsic currents also undergo changes that increase neuronal excitability. In acute conditions, the relative number of intrinsically bursting neurons doubles both in the undercut cortex and in surrounding areas (Topolnik et al., 2003a), which was likely induced by local changes of K^+^ concentration due to direct neuronal damage (Jensen et al., 1994; Jensen and Yaari, 1997). The membrane potential of neurons is more hyperpolarized within the undercut cortex compared to surrounding areas (Topolnik et al., 2003a). The neuronal excitability and the overall firing, particularly in deep layers, is decreased within the undercut cortex (Topolnik et al., 2003a). Overall this results in a misbalance in excitability in the undercut and surrounding areas, creating conditions for the generation of acute seizures. Indeed, we found, using detailed conductance based models of the thalamocortical network including Na^+^ and K^+^ ion concentration dynamics, that change in the normal balance of ionic concentrations (particularly an increase in extracellular K^+^ concentrations) may promote epileptiform discharges and lead to PDS (Frohlich et al., 2010; Krishnan and Bazhenov, 2011).

In chronic conditions the signs of changes of intrinsic excitability are opposite. Starting from 2 weeks after isolation in *in vivo* conditions the input resistance of neurons as well as their intrinsic excitability, which is measured as the number of spikes elicited by a given current pulse or as instantaneous firing rate, is increased (Avramescu and Timofeev, 2008). Similar finding was obtained *in vitro* (Prince and Tseng, 1993), suggesting that it is the network excitability and not necessarily the network properties of the traumatized tissue that is affected. Altogether the changes in intrinsic and synaptic excitability produce an increase in the duration of the silent state and a compensatory increase in the instantaneous spontaneous firing rates (*R* = 0.87, *p* < 0.01), suggesting that a homeostatic regulation of the neuronal excitability took place (Avramescu and Timofeev, 2008).

## HOMEOSTATIC PLASTICITY IN BRAIN TRAUMA RECOVERY AND EPILEPTOGENESIS

Brain excitability is maintained at a level via homeostatic mechanisms that is neither too low nor too high. Silencing a cortical culture network for 2 days upregulates synaptic excitability and an increase in network activity down-regulates excitatory synaptic efficacy (Turrigiano et al., 1998; Watt et al., 2000; Murthy et al., 2001), but not all connections (Kim and Tsien, 2008). Conversely, prolonged levels of enhanced activity induced by the blockade of synaptic inhibition or elevated [K^+^]_o_, reduces the size of mEPSCs (Lissin et al., 1998; Turrigiano et al., 1998; Leslie et al., 2001). Similar activity-dependent changes in mEPSC size have been observed in spinal cell cultures (O’Brien et al., 1998). Synaptic scaling occurs post-synaptically in part by changes in the number of open channels (Turrigiano et al., 1998; Watt et al., 2000), although all synaptic components may increase (Murthy et al., 2001) including numbers of postsynaptic glutamate receptors (Rao and Craig, 1997; Lissin et al., 1998; O’Brien et al., 1998; Liao et al., 1999). There is a similar regulation of NMDA currents by activity (Watt et al., 2000; see however Lissin et al., 1998). Interestingly, mIPSCs are scaled down with activity blockade, opposite in direction to changes in excitatory currents. This effect is reversible (Rutherford et al., 1997) and is accompanied by a reduction in the number of opened GABA_A_ channels and GABA_A_ receptors clustered at synaptic sites (Kilman et al., 2002). In addition, intrinsic excitability is regulated by activity. After chronic blockade of activity, Na^+^ currents increase and K^+^ currents decrease in size, resulting in an enhanced responsiveness of pyramidal cells to current injection (Desai et al., 1999). Some of these processes may also occur *in vivo* (Desai et al., 2002). Thus, homeostatic plasticity also controls the levels of neuronal activity through intrinsic mechanisms (Turrigiano et al., 1998; Murthy et al., 2001). In recent studies, we have demonstrated that (a) during TIE, cortical neurons undergo long-lasting silent periods during all states of vigilance (Nita et al., 2007), (b) in a neocortical kindling model of epilepsy, seizures are followed by continuing outlasting activity (Nita et al., 2008a, b). This outlasting activity can last for up to 2 h and consist of silent and active states. Therefore, silent periods are increased in both models of epileptogenesis.

Based on the experimental data, we developed network computational models in which partial cortical deafferentation led to up-regulation of the neuronal excitability and the development of seizure-like activity (Houweling et al., 2005; Frohlich et al., 2006, 2008; Volman et al., 2011a, b, 2012, 2013). First, we found that only sufficiently strong deafferentation leads to the pathological network synchronization; after a weak deafferentation homeostatic plasticity was able to recover the normal asynchronous network activity (Houweling et al., 2005). Therefore, we predicted the existence of a critical degree of deafferentation (a threshold) for pathological network reorganization. Second, we found that both spatially defined (Houweling et al., 2005) and randomly deafferented group of neurons may lead to pathological bursting (Frohlich et al., 2008). Third, we found that the network, to be prone to paroxysmal bursting should include a population of cells with relatively high density of intact neurons and a population of cells with high levels of deafferentation and low spontaneous activity (Volman et al., 2011a, b). This suggests that, in the heterogeneous networks, epileptic activity should arise near the boundary of intact and deafferented areas and propagate to the deafferented population as observed experimentally (Topolnik et al., 2003b; Nita et al., 2006, 2007). Fourth, our studies predicted a critical role of interaction between neurons and glial cells in TIE (Volman et al., 2012, 2013). More recently we developed a sophisticated network model implementing both homeostatic plasticity and ion concentration dynamics (Gonzalez et al., 2013). This study revealed that the threshold between normal and pathological network activity (Frohlich et al., 2010) is reduced after deafferentation followed by homeostatic scaling. Therefore, after deafferentation even physiological level fluctuations of the input to the network may trigger transition to recurrent epileptiform activity that would be impossible in the normal (healthy) network. 

Importantly, our modeling studies suggest an existence of bistability between normal and pathological (paroxysmal) activity in the same network depending on its initial connectivity structure. Homeostatic scaling can lead to the different dynamical network states depending on the initial connectivity: either to recovery of normal activity (when the damage was small) or pathological paroxysmal activity (when the damage was large).

### WHY DO NOT ALL ANIMALS DISPLAY DEAFFERENTATION-INDUCED EPILEPTOGENESIS?

We have conducted many experiments in which cortical undercut was used to trigger epileptogenesis. Most of the anesthetized cats presented acute seizure activity that stopped after several hours (Topolnik et al., 2003a, b), but only 65% of cats developed seizures in chronic conditions (Nita et al., 2006, 2007; Nita and Timofeev, 2007). These animals weighted more than 2.5 kg and their age was unknown. Since 2008, new regulations of the Canadian Council on Animal Care recommended that all experiments be performed on animals bred for research. Breeders sell only young cats (8–14 month), weighing only 2.0–2.5 kg. Since 2008 experiments on the partial deafferentation model of epileptogenesis were performed only on young cats. Given recent technological advances we now record electrographic activity immediately after the end of surgery, using a wireless system (Grand et al., 2013).

**FIGURE 4:**
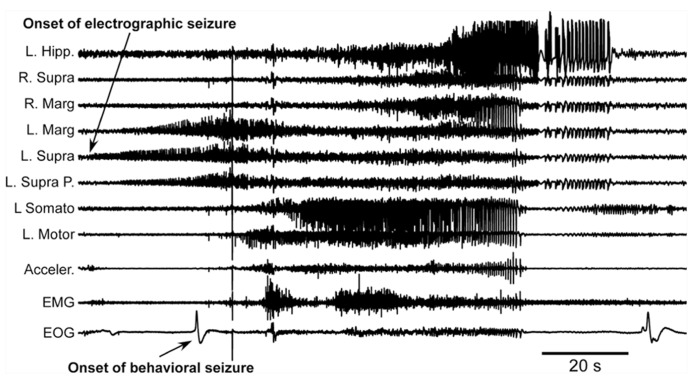
**Acute seizure recorded 8 h after the undercut and 6 h after the end of anesthesia.** Electrographic seizure starts in the anterior part of the suprasylvian gyrus and then propagates to other cortical areas and hippocampus. Behavioral seizure starts with eye movement and later with muscle contractions. Muscle activity occurs when motor cortex displays electrographic seizure. L, left; R, right; P, posterior; Hipp, hippocampus; Supra, suprasylvian gyrus; Marg, marginal gyrus; Somato, somatosensory cortex; Motor, motor cortex; Acceler, accelerometer; EMG, electromyogram; EOG, electrooculogram.

The first major finding was that, as in previous experiments, cats developed acute seizures (**Figure 4**). These seizures started at the border between the undercut and intact cortex [anterior part of the left suprasylvian gyrus (L. Supra) in **Figure 4**] and then other cortical areas became involved. Behavioral seizures started tens of seconds later. In the example shown in **Figure 4**, the behavioral seizure started with eye deflection, which happened 25 s after the onset of electrographic seizure. About 5 s later, the motor cortex got involved in paroxysmal activity and at the same time the behavioral seizure was also detected with electromyography (EMG) electrodes and accelerometer. Ipsilateral hippocampus and contralateral cortical electrodes showed involvement into seizure activity somewhere in the middle part of the entire seizure. Motor seizure detected with EMG electrodes and accelerometer stopped simultaneously with LFP paroxysmal activity in the motor cortex. The electrographic seizure remained ongoing in the hippocampus and several other cortical areas for another 20 s. The end of the seizure was characterized by a major eye movement. The seizure depicted in the **Figure 4** occurred 12 h after the undercut and 6 h after the end of anesthesia. Because these recordings were made after the end of anesthesia, and our previous investigations of acute seizures were done in cats anesthetized with ketamine–xylazine (Topolnik et al., 2003a, b; Avramescu and Timofeev, 2008), we concluded that anesthesia was not a factor leading to seizure development. These types of seizures stopped within 48 h from the undercut, suggesting that they were elicited by acute conditions created by the tissue damage (increased extracellular concentration of K^+^, glutamate, and other immediate actors of brain damage) but probably not by tissue reorganization.

**FIGURE5:**
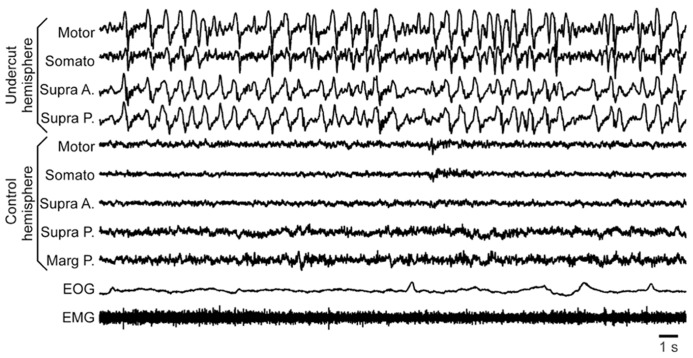
**Increased slow-wave activity in a partially deafferented hemisphere.** All abbreviations are as in **Figure 4**.

The second major finding was that in semi-chronic and chronic conditions the young cats showed an increase in slow-wave activity, mainly in the undercut cortex, but also in neighboring locations of the same hemisphere (**Figure 5**). The potential epileptogenesis was investigated in nine young cats (age 10–14 months at the time of surgery). Experiments on three of them were done in head-restrained conditions similar to previous experiments on adult cats (Nita et al., 2007) and the remaining six animals were freely moving. In freely moving cats, we used 24 h per day wireless recordings accompanied with continuous video recordings (Grand et al., 2013). The recordings lasted between 2 and 4 months. In chronic conditions, none of these animals showed any sign of epileptic activity: neither electrographic nor behavioral. Given our previous database, the absence of seizures after four initial experiments was surprising. Because these experiments were done on bred cats, we thought that their genetic background might prevent these animals to develop epileptogenesis. Therefore the other five cats for these experiments were bought from a different breeder to ensure a different genetic background. None of these animals developed epilepsy either. All rhythmic movements recorded in these animals were physiological: scratching, chowing, walking, etc. These movements on occasion were synchronized with a maximum of one LFP channel, normally located within motor or somatosensory cortical areas. In all these animals, the slow-wave activity was increased above undercut area, and slow waves appeared at all states of vigilance, although more rarely in a wake state and more often in a SWS state. Often, but not always, slow waves were enhanced in the whole undercut hemisphere during quiet wakefulness (**Figure 5**).

We need to explain a puzzling set of observations: (i) partial cortical deafferentation induces acute seizures in almost all investigated cats both under anesthesia and without anesthesia; (ii) partial cortical deafferentation results in chronic seizures in 65% of cats of unknown age, and (iii) partial cortical deafferentation does not trigger epileptogenesis and as a result, chronic seizures in young animals. Clearly the presence of acute seizures was not a sufficient factor to trigger epileptogenesis.

Reduced network activity (undercut, tetrodotoxin, or other) upregulates neuronal excitability via both intrinsic and synaptic mechanisms. The exact mechanisms are multiple, including increased density of sodium channels, axonal sprouting, increased neurotransmitter release, increased number of postsynaptic receptors, etc. *However, we hypothesized that in young animals there is a second-order mechanism that prevents the compensatory up-regulation of excitability to go to a pathological level. This conclusion is supported by multiple studies of homeostatic plasticity on either cultures or young animals (Turrigiano, 2011). In adult animals , at least partial deafferentation also leads to an increase in both intrinsic and synaptic excitability via multiple mechanisms (Avramescu and Timofeev, 2008; Avramescu et al., 2009). We propose that in adult animals the upper sensor, the one that evaluates at which level the increase in excitability needs to be stopped is not present or malfunctions, which leads to overt hyperexcitation and the onset of seizures.*

**FIGURE 6:**
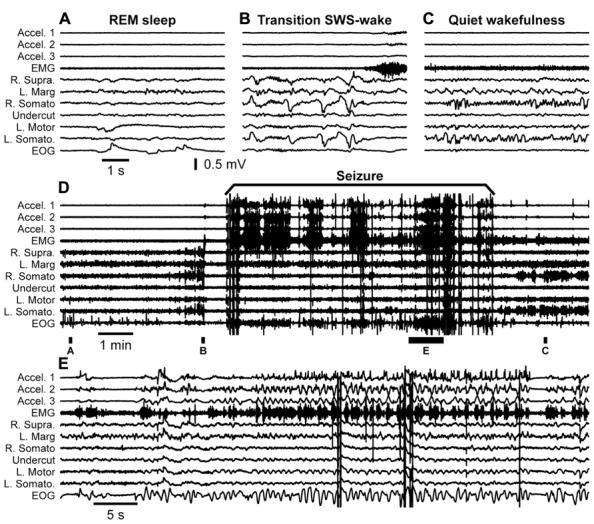
**Spontaneous seizure recorded in a cat 3.5 months after partial deafferentation of the suprasylvian gyrus.** The seizure occurred during quiet wakefulness. The full extent of seizure as well as pre and post seizure states of the brain is shown in the panel **(D)**. Panels **(A–C,E)** are segments expended in time as indicated in panel **(D)**. All abbreviations are as in **Figure 4**.

Our first step to confirm this hypothesis was to obtain an adult cat from the first breeder described above. The breeder received the cat 7 years ago as an adult and used it for reproduction. We performed undercut underneath the suprasylvian gyrus on this animal. Similar to other cats it had some acute seizures that stopped after 3 days from the undercut. Mild electrographic paroxysmal discharges reappeared 20 days after the surgery and their intensity progressively increased over several weeks. At 1.5 month from the partial cortical deafferentation the first behavioral seizures started. **Figure 6** shows an example full seizure that occurred 3.5 months from the undercut. These seizures usually started during quiet wakefulness. In the example shown in **Figure 6** the cat had a period of long REM sleep (**Figure 6A**), followed by a brief (40 s) period of SWS (**Figure 6B**) and followed by a brief (40 s) period of waking state. Awakening was characterized by postural adjustment (**Figure 6B**), but thereafter the animal did not move, suggesting that this was a period of quiet wakefulness. The onset of seizure was characterized by several major deflections on all recorded channels (**Figure 6D**), but thereafter the motor components of seizure recorded with accelerometers, EMG, and electrooculography (EOG) electrodes were accompanied with low amplitude phase locked LFP deflections in motor and visual cortices (**Figure 6E**). The seizure lasted for about 8 min. After the end of the seizure, the cat again went into a quiet state of wakefulness characterized by intense mu-rhythm activity recorded in the somatosensory cortices of both hemispheres.

Our studies suggest that various network properties, including the rate of homeostatic scaling and specifics of the neuronal glial interaction may affect the network susceptibility to epileptiform activity after homeostatic scaling triggered by deafferentation. We propose that some of these properties can change during development making adult animals more prone to seizures. The exact origin of these differences is still an open question that needs to be investigated.

## CONCLUSION

**FIGURE 7:**
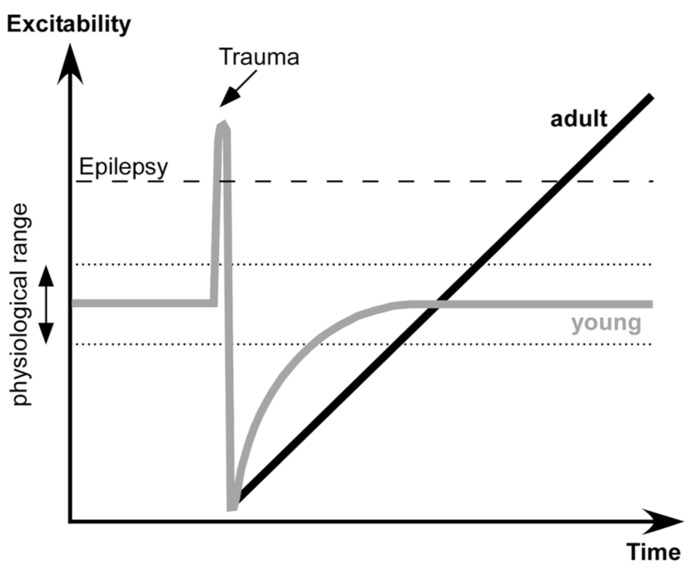
**Proposed dynamics of network excitability induced by brain trauma in young and adult subjects**.

The proposed dynamics of neuronal excitability produced by penetrating wounds are shown in the **Figure 7**. Acute trauma often elicits acute seizures that last for several hours. These seizures are not formally epileptic because they are produced as normal brain responses to the immediate sequelae of trauma: increased levels of extracellular K^+^, glutamate and other immediate factors of brain damage. Penetrating brain wounds reaching white matter also deafferent cortical regions. During normal brain operations, the excitation and inhibition are balanced (Shu et al., 2003; Haider et al., 2006) with a bias toward inhibition (Rudolph et al., 2007). Deafferentation removes a number of excitatory inputs to the affected cortical area, which can no longer maintain prolonged active states characterizing waking state and REM sleep.

Several physiological changes occur following deafferentation. During SWS the silent, hyperpolarized states become longer. A large number of neurons degenerate, particularly in deep cortical layers cortex (Avramescu et al., 2009), and in humans the onset of spontaneously active states in epileptic patients shifts toward more superficial layers (Cash et al., 2009). Increased network silence triggers a set of processes that upregulate cellular and network excitability that bring the deafferented neocortex to a normal level of excitability.

Here we propose that in young animals there is another set of processes that controls the extent of up-regulation and excitability: when physiological levels of excitability are attained, the up-regulation processes are stopped. However, in adult animals the sensor(s) for the upper limit of excitability is reduced or absent. Thus the up-regulation of network excitability becomes uncontrollable and the cortex becomes epileptic. The exact nature of this upper sensor is unknown, but it can share some known mechanisms of age-related cognitive impairment (Samson and Barnes, 2013). It can be an age-dependent altering of cyclic adenosine monophosphate (cAMP) signaling that controls neuronal excitability via hyperpolarization-activated cyclic nucleotide (HCN) or KCNQ channels (Wang et al., 2011), age-dependent changes in a control of inhibitory activities (Bories et al., 2013), changes in action potential properties (Luebke and Chang, 2007), change in a control of synaptic excitability in upper layer neurons (Luebke et al., 2004), or other changes.

Current therapies try to control seizures by increasing GABA inhibition or reducing neuronal excitability by blocking Na^+^ channels. In view of the evidence presented here that silencing cortical activity may trigger epileptogenesis, these treatments may be counterproductive. Epileptogenesis is a latent period that has not yet been the target for antiepileptic therapy. The first step is to identify the mechanisms for the deafferentation-induced up-regulation of neuronal excitability. We have proposed that the controller in young animals that limits normal excitability is absent in adults. If it is possible to upregulate this controller and partially recover the normal network function in the deafferented region in animal models, the same intervention may also mitigate the development of trauma-induced epilepsy in adult humans.

## Conflict of Interest Statement

The authors declare that the research was conducted in the absence of any commercial or financial relationships that could be construed as a potential conflict of interest.

## Abbreviations

cAMP: cyclic adenosine monophosphate
EEG: electro-encephalography
EMG: electromyography
EOG: electrooculography
EPSC: excitatory postsynaptic current
EPSP: excitatory postsynaptic potential
HCN: hyperpolarization-activated cyclic nucleotide-gated
IPSC: inhibitory postsynaptic current
IPSP: inhibitory postsynaptic potential
KCNQ: delayed rectifier voltage-gated potassium channel
LFP: local field potential
PDS: paroxysmal depolarizing shift
REM: rapid eye movement
SWS: slow-wave sleep
TIE: trauma-induced epileptogenesis
W: wake
